# From the Editor‐in‐Chief

**DOI:** 10.1111/pbi.14560

**Published:** 2024-12-26

**Authors:** Johnathan Napier

As 2024 draws to a close, it once again provides an opportunity to recognize and thank everyone who has contributed to the continued success of *Plant Biotechnology Journal*, a combined effort from the Editors, the reviewers and the Wiley team. I deeply appreciate the diligence and hard work everyone puts into helping authors elevate and improve their manuscripts, ultimately providing useful additions to the published literature.

*Plant Biotechnology Journal* continues to be a top‐ranked journal, maintaining its position in the top five titles in the Plant Sciences category. Our Editors have handled over 1300 submissions so far this year, and we are on track to publish ~250 papers in 2024. It is fair to say that the PBJ team and I are very proud of our metrics and rankings (Impact Factor 11.2 and CiteScore 20.5). Perhaps, a more impressive number is for article downloads, which for 2024 was ~2 million—to my mind, that really does demonstrate relevance and utility. In 2024, PBJ also provided sponsorship to a diverse range of scientific meetings, helping to build communities and forge new connections. A personal highlight for me was the recent meeting in Samson, Turkey entitled *Agricultural Biotechnology in the Era of Genome Editing* which was organized by AAB and supported by PBJ. I was incredibly impressed with the science presented at this congress as well as the enthusiasm of the participants—I felt lucky to be part of it.

Given the global challenges that we face, the importance of discovery and innovation is ever more apparent and necessary. Although the massive disruption of Covid‐19 now seems like a distant memory, it is important to recall that it was scientific innovation (in the form of new vaccines) that stemmed the pandemic. Now we face probably even greater, more complex threats in the form of accelerating climate change and the associated disruption to environments and food production. I believe that plant biotechnology (and PBJ as part of that vibrant community) can play a key role in mitigating these perturbations, and I would encourage everyone to consider how their knowledge and expertise might contribute solutions to these global challenges. Plant biotechnology has delivered amazing disruptive agricultural innovations at a massive scale (such as herbicide‐tolerant crops), and I believe that it can and will do so again in response to the climate crisis. In that respect, PBJ will continue to play a role, publishing the best and most useful advances in our field.

Best wishes for 2025,

Johnathan

